# Child Aflatoxin Exposure is Associated with Poor Child Growth Outcomes: A Prospective Cohort Study in Rural Malawi

**DOI:** 10.1016/j.cdnut.2023.101962

**Published:** 2023-06-10

**Authors:** Andrew Matchado, Joshua W. Smith, Kerry J. Schulze, John D. Groopman, Emma Kortekangas, David Chaima, Charles D. Arnold, Kenneth Maleta, Ulla Ashorn, Per Ashorn, Kathryn G. Dewey, Christine P. Stewart

**Affiliations:** 1 School of Global and Public Health, Kamuzu University of Health Sciences, Blantyre, Malawi; 2 Department of Environmental Health and Engineering, Bloomberg School of Public Health, Johns Hopkins University, Baltimore, MD, United States; 3 Department of International Health, Center for Human Nutrition, Bloomberg School of Public Health, Johns Hopkins University, Baltimore, MD, United States; 4 Center for Child, Adolescent, and Maternal Health Research, Tampere University and Tampere University Hospital, Tampere, Finland; 5 Department of Nutrition, Institute for Global Nutrition, University of California, Davis, CA, United States; 6 Department of Paediatrics, Tampere University Hospital, Tampere, Finland

**Keywords:** aflatoxin, AF B_1_-lysine adduct, anthropometric outcome, child growth, Malawi

## Abstract

**Background:**

Aflatoxin (AF) exposure is associated with child growth faltering in cross-sectional studies, with limited findings from longitudinal studies.

**Objectives:**

To evaluate the relationship between maternal AF B_1_-lysine adduct concentration, child AF B_1_-lysine adduct concentration, and child growth in the first 30 mo of life.

**Methods:**

AF B_1_-lysine adduct was measured in mother-child dyad plasma samples using isotope dilution mass spectrometry. Using linear regression, we assessed the relationship between AF B_1_-lysine adduct concentration and child weight, height, and head and mid-upper arm circumferences at 1 wk, 6, 12, 18, 24, and 30 mo of age.

**Results:**

In adjusted models, maternal prenatal AF B_1_-lysine adduct (pg/μL) was positively associated with newborn anthropometric outcomes; largest beta coefficients for associations between standardized values were for newborn weight-for-age *z*-score [β = 0.13; 95% confidence interval (CI): 0.02, 0.24; *P* < 0.05 and β = 0.11; 95% CI: 0.00, 0.22; *P* < 0.05 for second and third trimester AF, respectively]. Child AF B_1_-lysine adduct (pg/μL) at 6 mo was negatively associated with head circumference-for-age *z*-score at 6, 18, 24, and 30 mo, with beta coefficients ranging from β = –0.15; 95% CI: –0.28, –0.02 to β = –0.17; 95% CI: –0.31, –0.03; *P* < 0.05); 18-mo AF was negatively associated with anthropometric outcomes at 18, 24, and 30 mo, most consistently with length-for-age *z*-score (β = –0.18; 95% CI: –0.32, –0.04, β = –0.21; 95% CI: –0.35, –0.07, β = –0.18; 95% CI: –0.32, –0.03 at 18, 24 and 30 mo, respectively).

**Conclusions:**

Child AF exposure was associated with impaired child growth, but maternal AF exposure was not. Exposure during infancy was linked to persistent deficit in head circumference, implying reduced brain size lasting beyond the age of 2 years. Exposure at 18 mo was linked to persistent linear growth deficit. Further research should elucidate mechanisms through which AF affects child growth.

## Introduction

Aflatoxins (AFs) are mycotoxins produced by the fungi *Aspergillus flavus* and *Aspergillus parasiticus*. The AFs include AFB_1_, AFB_2_, AFG_1_, and AFG_2_, and of these, AFB_1_ is the most toxic [1,2]. Chronic exposure to AF is associated with increased risk of primary liver cancer in humans [3], and exposure during pregnancy is associated with adverse birth outcomes as well as linear growth retardation during infancy [[4], [5], [6], [7], [8], [9], [10], [11]]. Maize and groundnuts are the most predominant foods contaminated with AF, particularly when storage conditions and inadequate drying of crops promote growth of the toxin-producing fungi [1,2]. Individuals living in low-and-middle-income countries where these crops are staple foods are at greatest risk of exposure [12].

AF exposure in humans generally occurs through ingestion of contaminated foods, including cooked foods [13,14]. AF exposure can occur as early as in utero because AF can cross the placental barrier during pregnancy [4]. Once absorbed in the small intestine, AFs are transformed by enzymes in the liver to the highly reactive and toxic metabolite AFB_1_-*exo*-8, 9-epoxide [15,16]. The epoxide can bind with cellular macromolecules like DNA and protein, forming AF B_1_-N7-guanine adducts and serum AF B_1_-lysine adducts [17]. These metabolites are found in urine and blood, respectively. Although metabolites in urine can be used as biomarkers of recent (∼24 h) exposure to AF [18], plasma AF B_1_-lysine adduct is the biomarker of choice for assessing exposure across months because of the long half-life of serum albumin in circulation [17].

To date, few longitudinal studies have assessed the association between AF exposure and child growth outcomes, and the few that have done so have yielded inconsistent results [19,20]. We recently reported nearly 100% prevalence of persistently high AF exposure among pregnant and lactating women and their young children in Malawi (Supplementary Table 1). Persistently high AF exposure was defined as high AF exposure (AFB_1_-lysine adduct concentration ≥0.44 pg/μL) assessed in the second and third trimesters and at 6 mo postpartum [21]. An exception to the high prevalence of exposure observed in this population was among infants at 6 mo of age, in whom exposure prevalence was 60%. Additionally, we reported a strong correlation between maternal and child AFB_1_-lysine concentrations measured in samples collected from the mothers at 6 mo after delivery and in the children at 6 mo of age (ρ = 0.63; *P* < 0.0001) [21]. The objective of this longitudinal study, a follow-up to the previously reported study, was to determine whether maternal or child plasma AFB_1_-lysine adduct concentrations are related to child growth outcomes.

## Methods

### Study design and participants

This was a substudy of the iLiNS-DYAD (international lipid-based nutrient supplements) trial that was conducted in Mangochi district, Malawi, from February 2011 to April 2015 [22]. The iLiNS-DYAD trial was approved by the institutional review board of the University of California, Davis, and the ethics committees of Kamuzu University of Health Sciences; and the Pirkanmaa Hospital District, Finland. The trial assessed whether small-quantity lipid-based nutrient supplements consumed by the mother during pregnancy and the first 6 mo of lactation, and by the child from 6–18 mo, improve fetal and child growth, micronutrient status, and neuro-behavioral development to a greater extent than consumption of iron and folic acid during pregnancy only, or multiple micronutrient tablets during pregnancy and the first 6 mo of lactation. A total of 1391 pregnant women were enrolled at ≤20 completed gestation weeks. The sample size was based on an assumption of an effect size of ≥0.3 (difference between groups, divided by the pooled SD) for each continuous outcome, a power of 80%, and a 2-sided type I error rate of 5%. Inclusion and exclusion criteria for the trial were published previously [22]. The current study included a subset of 255 mother-child dyads from the trial for whom we had plasma samples collected at enrollment, 36 weeks of gestation, and 6 mo postpartum for the women and at 6 and 18 mo for their offspring, and the offspring had complete anthropometric data.

### Anthropometric assessment

At enrollment, anthropometrists measured the mother’s weight, height, and MUAC using high-quality scales (SECA 874 flat scale; Seca GmbH & Co.), stadiometers (Harpenden stadiometer; Holtain Limited), and nonstretchable plastic tapes (Shorrtape; Weigh and Measure, LLC) with reading increments of 50 g, 1 mm, and 1 mm, respectively. The infant’s birth weight was measured as soon as possible after birth or within 2 wk after birth, and a more thorough postnatal infant visit was completed when the infant was 1–6 wk old at the study clinic. Infant length was measured with a high-quality length board (Harpenden Infantometer; Holtain Limited) and recorded to the nearest 1 mm, weight was measured with an electronic infant weighing scale with a reading increment of 20 g (SECA 381 baby scale; Seca GmbH & Co.), and head circumference and MUAC were measured with the same plastic tapes that were used for maternal anthropometry. Anthropometric assessments were also conducted at the study clinic when the children were 6, 12, 18, 24, and 30 mo old. Anthropometric measurements were completed in triplicate [22]. The mean value of the first 2 anthropometric measurements was used unless a prespecified difference was exceeded, in which case the mean of the 2 closest values was used. Age- and sex-standardized anthropometric indices (weight-for-age *z*-score [WAZ]; length-for-age *z*-score [LAZ]; weight-for-length *z*-score [WLZ]; MUAC-for-age *z*-score [MUACZ] and head circumference-for-age *z*-score [HCZ]) were created using the WHO Child Growth Standards [23].

### Analysis of AFB_1_-lysine adduct concentration

About 7.5 and 5 mL whole blood was collected from the mothers and children, respectively, into Sarstedt Monovette trace element-free tubes by venipuncture at enrollment, 36 weeks of gestation, and 6 mo postpartum for the mothers and at 6 and 18 mo of age for the children. The tubes contained heparin anticoagulant. The blood samples were centrifuged (3 min at room temperature at 14,000 × *g*), and plasma was stored at –80°C. We identified 255 mother-child dyads with archived plasma samples collected at enrollment, 36 weeks of gestation, and 6 mo postpartum for the women and at 6 and 18 mo for their offspring. The samples were analyzed for AFB_1_-lysine adduct concentration in the Groopman laboratory in the Department of Environmental Health and Engineering, Bloomberg School of Public Health, Johns Hopkins University, Baltimore, MD, United States.

The archived plasma samples were analyzed for AFB_1_-lysine adduct concentration using isotope dilution MS (IDMS) as previously described [21]. The limit of quantification for the assay was determined to be at 0.01 pg/μL, and values less than the limit of quantification were imputed at 0.005 pg/μL. AFB_1_-lysine adduct concentration presented in this study was not expressed relative to albumin. It has been reported elsewhere that normalizing AFB_1_-lysine adduct concentration to serum albumin is not necessary when the former was measured by IDMS [24]. We did human serum albumin (HSA) normalization in plasma samples of mothers (*n* = 98) collected at baseline and 36 weeks of gestation in this current study to see if normalization would make a difference. We compared paired albumin concentrations in the mothers across the 2 different time points using 2 different validated assays (QuantiChrom BCP, BioAssay Systems Inc. [bromocresol purple] assay, catalog #DIAP-250 and COBAS INTEGRA, Roche Diagnostics Albumin Gen.2 [ALB2] Test ALB2, test ID 0-59 BCG [bromocresol green] assay; BioAssay Systems Inc.). We found assay-dependent differences and very weak/no correlation between the values determined by the 2 albumin assays at either the baseline (r = 0.11; *P* = 0.26) or 36 weeks of gestation (r = 0.25; *P* = 0.012) time points (Supplementary Figure 1). Secondly, we found statistically significant quantitative biases between the 2 assays at both baselines (+11.9 mg/mL; *P* < 0.0001) and 36 weeks of gestation (+9.4 mg/mL; *P* < 0.0001). Finally, we found significant differences between the baseline and 36 weeks of gestation time points with 1 assay (–3.2 mg/mL; *P* < 0.0001), whereas there were no differences between time points when determined by the second assay (–0.7 mg/mL; *P* = 0.35). Normalization of AFB_1_-lysine adducts to serum total albumin did not improve AFB_1_ internal dose estimation.

### Covariates

Potential confounding factors were identified from scientific literature and included indicators of maternal anthropometry and weight gain, including weight at baseline, prepregnancy BMI (in kg/m^2^) (estimated from BMI at enrollment), and weight and MUAC gain rate from baseline to 36 weeks of gestation (difference between measurements at 36 wk and baseline divided by number of weeks in between). In addition to their likely association with infant growth, these variables may also serve as proxies of maternal EI and, thereby, consumption of the staple food, maize. Other maternal factors included maternal age, parity (primiparity compared with multiparity), hemoglobin (measured from a venous blood sample using HemoCue AB), zinc protoporphyrin (measured from washed RBCs with a hematofluorometer; Aviv Biomedical, Inc.), HIV status (measured using whole-blood antibody rapid test, Alere Determine HIV-1/2; Alere Medical Co, Ltd.) and malaria status at baseline (measured from a venous blood sample using the rapid test, Clearview Malaria Combo; British Biocell International Ltd.). Child factors included sex, age, hemoglobin, malaria status at 6 and 18 mo of age, porridge consumption, and predominant breastfeeding at 4 and 6 mo of age. Household factors included the household food insecurity access score and housing quality (by assessing if the house wall was built of brick and if roofing material was made of iron sheets). Finally, we also considered season of the outcome assessment (wet and dry) and intervention group (Supplementary Table 2).

### Statistical analysis

All analyses were done using SAS version 9.4, SAS Institute Inc. AFB_1_-lysine adduct concentration was log-transformed (log base 10) prior to analysis. Maternal AF exposure during pregnancy was assessed in relation to newborn anthropometric outcomes and child anthropometric outcomes measured from 6–30 mo of age. Postnatal maternal AF exposure and child AF exposure were assessed in relation to child anthropometric outcomes measured from 6–30 mo of age. We analyzed 2 separate linear regression models to assess the relationship between AFB_1_-lysine adduct concentration and child anthropometric outcomes: the first model was unadjusted, whereas the second model was adjusted for potentially confounding factors. Covariates related to each outcome variable with a *P* value of 0.10 in bivariate analysis were retained in the final model. The intervention group was included in all multivariable models regardless of its statistical significance. Additionally, for the child AF exposure at 6 mo, we modeled the explanatory variable as a categorical variable: detectable compared with nondetectable child AF. We tested for multicollinearity among the covariates by calculating the variance inflation factor for each independent variable. If multiple independent variables were collinear and represented a similar construct, the variable that explained the most variation in AF (as measured by R^2^) was included in the final multivariable model. AFB_1_-lysine adduct concentration was standardized to have a mean of 0 and SD of 1 for easy comparison of the variables to each other. All results were presented as beta coefficients with CI set at 95% and *P* values. A *P* value < 0.05 indicated statistical significance in both the unadjusted and adjusted models. The beta coefficients were interpreted as a standard change in the outcome as a result of a 1 SD change in the exposure.

## Results

**FIGURE 1:**
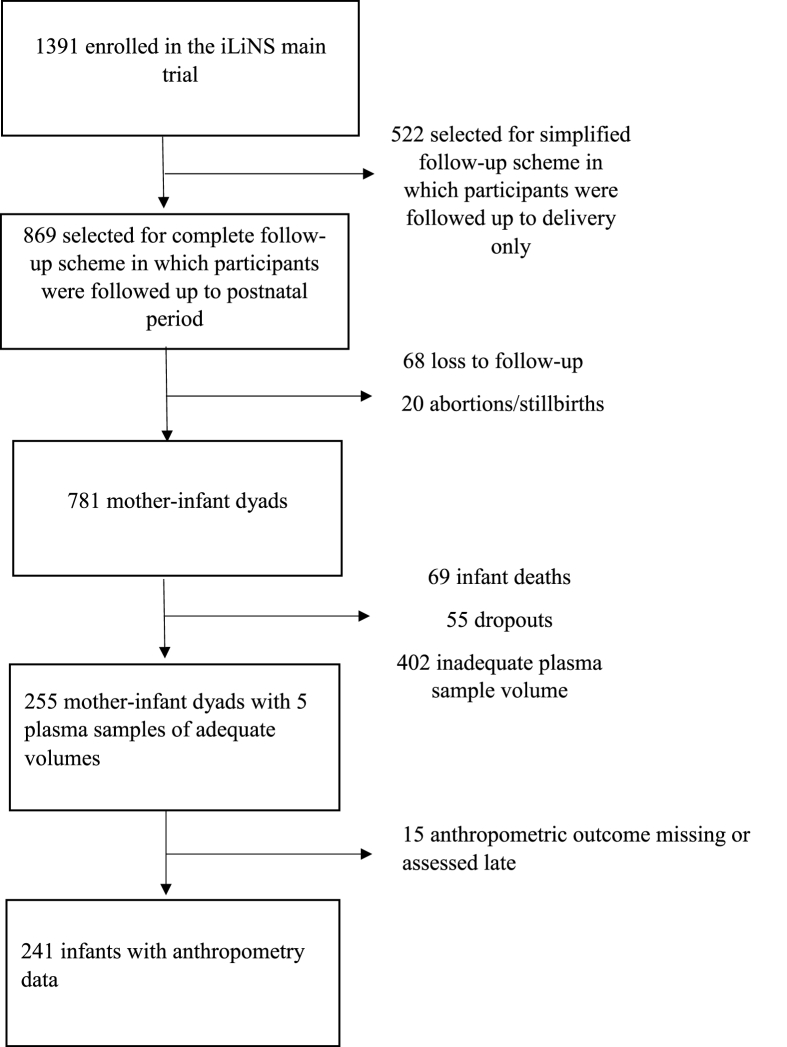
Study flow diagram. iLiNS, international lipid-based nutrient supplements.

Of the 255 mother-infant dyads with plasma samples at all 5-time points, 241 infants had complete anthropometric data (Figure 1). Overall, the participants included in the substudy did not differ in baseline characteristics from those in the main trial not included in this substudy (Table 1). At enrollment, the mean (SD) maternal age, prepregnancy BMI, gestational age, and years of education were 25 (6) y, 21.5 (2.5), 16.9 (2.3) wk, and 3.7 (3.4) y, respectively. More than 1-third (39%) of the households were severely food insecure.

**TABLE 1:** Baseline maternal characteristics of participants in the main international lipid-based nutrient supplements trial and those included in the substudy

Maternal characteristics	iLiNS-DYAD trial		Aflatoxin substudy		*P* value
	*N*	Mean (SD) or %	*N*	Mean (SD) or %	
Age (y)	1136	25 (6)	255	25 (6)	0.82
Prepregnancy BMI^1^ (kg/m^2^)	1118	21.8 (2.8)	252	21.5 (2.5)	0.08
Education (y)	1075	4.1 (3.5)	251	3.7 (3.4)	0.12
Primiparous (%)	1133	22.2	255	20.0	0.42
Gestational age at enrollment (wk)	1136	16.8 (2.1)	255	16.9 (2.3)	0.73
Hemoglobin (g/L)	1135	111.0 (16.0)	254	113.0 (16.0)	0.15
Piped water (%)	1074	16.0	254	12.6	0.15
Malaria, positive (%)	1136	23.6	252	21.4	0.45
HIV status, positive (%)	1080	14.3	254	11.4	0.21
Food security (%)	1072		251		0.05
Food secure		19.9		17.9	
Mildly food insecure		14.4		8.4	
Moderately food insecure		30.4		35.1	
Severely food insecure		35.4		38.7	
MUAC gain rate^2^ (cm/wk)	810	0.0 (0.1)	250	–0.01 (0.1)	0.06

iLiNS-DYAD, international lipid-based nutrient supplements.

1 Estimated using enrollment BMI.

2 MUAC gain rate = mid-upper arm circumference gain rate.

### Newborn anthropometric outcomes

Maternal AFB_1_-lysine adduct concentration during pregnancy was positively associated with newborn WAZ but not with the other birth measures (Table 2). The associations were generally stronger with the maternal baseline concentration than with the concentration at 36 weeks of gestation (β = 0.13; 95% CI: 0.02, 0.24; *P* < 0·05 and β = 0.11; 95% CI: 0.00, 0.22 *P* <0·05, respectively)

**TABLE 2:** Association between maternal aflatoxin B_1_-lysine adduct concentration and newborn length-for-age *z*-score, weight-for-age *z*-score, weight-for-length *z*-score and head circumference-for-age *z*-score at 1 wk [β^1^ (95% CI)], *n* = 234

	Baseline aflatoxin B_1_-lysine adduct		36 wk aflatoxin B_1_-lysine adduct	
	Unadjusted	Adjusted^2^	Unadjusted	Adjusted^2^
Anthropometric outcome at 1 wk	β (95% CI)	β (95% CI)	β (95% CI)	β (95% CI)
Length-for-age *z*-score	0.16 (0.03, 0.29)∗	0.12 (–0.01, 0.25)	0.12 (–0.01, 0.24)	0.10 (–0.02, 0.22)
Weight-for-age *z*-score	0.18 (0.06, 0.30)∗∗	0.13 (0.02, 0.24)∗	0.15 (0.03, 0.27)∗	0.11 (0.00, 0.22)∗
Weight-for-length *z*-score	0.07 (–0.07, 0.22)	0.07 (–0.07, 0.22)	0.10 (–0.04, 0.24)	0.10 (–0.04, 0.24)
Head circumference-for-age *z*-score	0.12 (0.00, 0.24)∗	0.11 (–0.01, 0.22)	0.09 (–0.03, 0.20)	0.09 (–0.02, 0.20)

1 β coefficient is the SD difference in outcome per SD difference in log aflatoxin

2 Adjusted for maternal age, prepregnancy BMI, parity, HIV, hemoglobin, weight and MUAC gain rate from baseline to 36 wk, season, housing quality, child sex, and age.

### Child growth outcomes measured from 6–30 mo of age

Maternal AFB_1_-lysine adduct concentration at baseline, 36 weeks of gestation, and at 6 mo postpartum was not significantly associated with child growth outcomes measured at 6, 12, 18, 24, or 30 mo of age (Supplementary Table 3 and Table 3). By contrast, child AFB_1_-lysine adduct concentration at 6 mo was consistently negatively associated with HCZ at 6 mo (β = –0.17; 95% CI: –0.31, –0.03; *P* < 0·05), 18 mo (β = –0.15; 95% CI: –0.28, –0.03; *P* < 0·05), 24 mo (β = –0.15; 95% CI: –0.28, –0.02; *P* < 0·05) and 30 mo (β = –0.16; 95% CI: –0.30, –0.02; *P* < 0·05) (TABLE 3, TABLE 4). In addition, it was negatively associated with WLZ at 24 mo (β = –0.14; 95% CI: –0.27, –0.02; *P* < 0·05). Child AFB_1_-lysine adduct concentration at 18 mo was negatively associated with concurrent LAZ (β = –0.18; 95% CI: –0.32, –0.04; *P* < 0·05), WAZ (β = –0.19; 95% CI: –0.32, –0.06; *P* < 0·05), WLZ (β = –0.13; 95% CI: –0.25, –0.06; *P* < 0·05), and MUACZ (β = –0.19; 95% CI: –0.30, –0.07; *P* < 0·01) (Table 4). Similarly, it was negatively associated with LAZ (β = –0.21; 95% CI: –0.35, –0.07; *P* < 0·01), WAZ (β = –0.17; 95% CI: –0.29, –0.04; *P* < 0·01) and MUACZ (β = –0.18; 95% CI: –0.30, –0.07; *P* < 0·01) at 24 mo of age, but only with LAZ (β = –0.18; 95% CI: –0.32, –0.03; *P* < 0·05) measured at 30 mo of age (Table 4).

**TABLE 3:** Association between aflatoxin B_1_-lysine adduct concentration and length-for-age *z*-score, weight-for-age *z*-score, weight-for-length *z*-score, MUAC-for-age *z*-score, head circumference-for-age *z*-score at 6 and 12 mo [β^1^ (95% CI)]

	Maternal aflatoxin B_1_-lysine adduct						Child aflatoxin B_1_-lysine adduct	
	Baseline		36 wk		6 mo postpartum		6 mo	
	Unadjusted	Adjusted^2^	Unadjusted	Adjusted^2^	Unadjusted	Adjusted^2^	Unadjusted	Adjusted^2^
	β (95% CI)	β (95% CI)	β (95% CI)	β (95% CI)	β (95% CI)	β (95% CI)	β (95% CI)	β (95% CI)
Anthropometric outcomes at 6 mo, *n* = 203								
LAZ	0.04 (–0.11, 0.18)	–0.01 (–0.17, 0.15)	0.07 (–0.07, 0.21)	0.07 (–0.09, 0.22)	–0.08 (–0.23, 0.07)	–0.05 (–0.21, 0.11)	–0.15 (–0.30, 0.01)	–0.11 (–0.26, 0.06)
WAZ	0.08 (–0.07, 0.23)	0.08 (–0.09, 0.24)	0.11 (–0.03, 0.25)	0.11 (–0.04, 0.27)	0.00 (–0.16, 0.15)	–0.05 (–0.21, 0.11)	–0.17 (–0.33, –0.01)∗	–0.16 (–0.33, 0.01)
WLZ	0.06 (–0.07, 0.20)	0.09 (–0.05, 0.22)	0.08 (–0.05, 0.22)	0.10 (–0.03, 0.24)	0.06 (–0.09, 0.20)	0.04 (–0.10, 0.18)	–0.10 (–0.25, 0.05)	–0.08 (–0.23, 0.07)
MUACZ	0.05 (–0.09, 0.19)	0.10 (–0.07, 0.26)	0.03 (–0.10, 0.16)	–0.03 (–0.19, 0.12)	–0.01 (–0.15, 0.14)	–0.02 (–0.19, 0.15)	–0.14 (–0.29, 0.01)	–0.07 (–0.25, 0.11)
HCZ	0.02 (–0.11, 0.16)	0.00 (–0.13, 0.13)	0.03 (–0.10, 0.16)	0.03 (–0.10, 0.16)	–0.11 (–0.25, 0.03)	–0.09 (–0.23, 0.04)	–0.20 (–0.35, –0.06)∗	–0.17 (–0.31, –0.03)∗
Anthropometric outcomes at 12 mo, *n* = 202								
LAZ	0.07 (–0.09, 0.22)	0.04 (–0.11, 0.19)	0.13 (–0.02, 0.27)	0.13 (–0.01, 0.28)	–0.03 (–0.19, 0.12)	–0.04 (–0.19, 0.11)	–0.15 (–0.30, 0.01)	–0.13 (–0.29, 0.03)
WAZ	0.04 (–0.11, 0.19)	0.05 (–0.10, 0.19)	0.09 (–0.05, 0.23)	0.11 (–0.04, 0.25)	0.02 (–0.12, 0.17)	0.00 (–0.15, 0.14)	–0.11 (–0.26, 0.05)	–0.10 (–0.25, 0.05)
WLZ	–0.01 (–0.15, 0.13)	0.02 (–0.11, 0.16)	0.02 (–0.11, 0.15)	0.06 (–0.07, 0.19)	0.05 (–0.09, 0.18)	0.03 (–0.11, 0.16)	–0.05 (–0.20, 0.09)	–0.06 (–0.20, 0.08)
MUACZ	0.04 (–0.10, 0.17)	0.05 (–0.09, 0.18)	0.09 (–0.04, 0.22)	0.09 (–0.04, 0.23)	0.10 (–0.04, 0.23)	0.09 (–0.04, 0.23)	–0.04 (–0.17, 0.10)	–0.02 (–0.17, 0.12)
HCZ	–0.06 (–0.19, 0.08)	–0.06 (–0.19, 0.08)	–0.02 (–0.14, 0.11)	–0.02 (–0.14, 0.11)	–0.05 (–0.18, 0.08)	–0.05 (–0.18, 0.08)	–0.11 (–0.24, 0.03)	–0.11 (–0.24, 0.03)

HCZ, head circumference-for-age *z*-score; LAZ, length-for-age *z*-score; MUACZ, mid-upper-arm-circumference-for-age *z*-score; WAZ, weight-for-age *z*-score; WLZ, weight-for-length *z*-score.

∗∗∗*P* < 0·001, ∗∗*P* < 0·01, ∗*P* <0·05.

1 β coefficient is the SD difference in outcome per SD unit difference in log aflatoxin.

2 Adjusted for maternal prepregnancy BMI, zinc protoporphyrin, weight and MUAC gain rate from baseline to 36 wk, housing quality, season, child sex, age, hemoglobin, porridge consumption at 4 and 6 mo for outcomes at 6 mo; adjusted for maternal prepregnancy BMI, HIV, weight and MUAC gain rate from baseline to 36 wk, housing quality, season, child sex, and age for outcomes at 12 mo.

**TABLE 4:** Association between child aflatoxin B_1_-lysine adduct concentration and length-for-age *z*-score, weight-for-age *z*-score, weight-for-length *z*-score, MUAC-for-age *z*-score, and head circumference-for-age *z*-score at 18, 24, 30 mo [β^1^ (95% CI)]

	Child aflatoxin B_1_-lysine adduct			
	6 mo		18 mo	
	Unadjusted	Adjusted^2^	Unadjusted	Adjusted^2^
	β (95% CI)	β (95% CI)	β (95% CI)	β (95% CI)
Anthropometric outcomes at 18 mo, *n* = 207				
LAZ	–0.09 (–0.24, 0.05)	–0.08 (–0.23, 0.06)	–0.14 (–0.28, 0.01)	–0.18 (–0.32, –0.04)∗
WAZ	–0.12 (–0.25, 0.01)	–0.13 (–0.26, 0.01)	–0.16 (–0.29, –0.03)∗	–0.19 (–0.32, –0.06)∗
WLZ	–0.10 (–0.22, 0.03)	–0.09 (–0.21, 0.04)	–0.13 (–0.25, –0.01)∗	–0.13 (–0.25, –0.01)∗
MUACZ	–0.07 (–0.19, 0.06)	–0.05 (–0.17, 0.07)	–0.16 (–0.29, –0.04)∗	–0.19 (–0.30, –0.07)∗∗
HCZ	–0.14 (–0.27, –0.01)∗	–0.15 (–0.28, –0.03)∗	–0.13 (–0.25, 0.00)∗	–0.10 (–0.22, 0.03)
Anthropometric outcomes at 24 mo, *n* = 187				
LAZ	–0.03 (–0.18, 0.11)	–0.03 (–0.18, 0.11)	–0.18 (–0.31, –0.04)∗	–0.21 (–0.35, –0.07)∗∗
WAZ	–0.11 (–0.24, 0.03)	–0.12 (–0.25, 0.01)	–0.11 (–0.24, 0.02)	–0.17 (–0.29, –0.04)∗∗
WLZ	–0.12 (–0.26, 0.01)	–0.14 (–0.27, –0.02)∗	–0.03 (–0.16, 0.09)	–0.07 (–0.19, 0.05)
MUACZ	–0.08 (–0.21, 0.05)	–0.12 (–0.24, 0.00)	–0.14 (–0.26, –0.02)∗	–0.18 (–0.30, –0.07)∗∗
HCZ	–0.14 (–0.27, –0.01)∗	–0.15 (–0.28, –0.02)∗	–0.10 (–0.22, 0.03)	–0.12 (–0.24, 0.01)
Anthropometric outcomes at 30 mo, *n* = 179				
LAZ	–0.07 (–0.22, 0.08)	–0.07 (–0.22, 0.08)	–0.15 (–0.28, –0.01)∗	–0.18 (–0.32, –0.03)∗
WAZ	–0.07 (–0.21, 0.06)	–0.06 (–0.19, 0.08)	–0.04 (–0.16, 0.09)	–0.03 (–0.17, 0.10)
WLZ	–0.02 (–0.16, 0.11)	–0.02 (–0.15, 0.12)	0.06 (–0.07, 0.18)	0.09 (–0.04, 0.22)
MUACZ	–0.04 (–0.17, 0.09)	–0.03 (–0.16, 0.10)	–0.07 (–0.19, 0.05)	–0.02 (–0.15, 0.10)
HCZ	–0.18 (–0.31, –0.04)∗	–0.16 (–0.30, –0.02)∗	–0.12 (–0.25, 0.01)	–0.08 (–0.21, 0.06)

HCZ, head circumference-for-age *z*-score; LAZ, length-for-age *z*-score; MUACZ, mid-upper-arm-circumference-for-age *z*-score; WAZ, weight-for-age *z*-score; WLZ, weight-for-length *z*-score.

∗∗∗*P* < 0·001, ∗∗*P* < 0·01, ∗*P* < 0·05.

1 β coefficient is the SD difference in the outcome per SD difference in log aflatoxin

2 Adjusted for maternal prepregnancy BMI, HIV, weight and MUAC gain rate from baseline to 36 wk, housing quality, food security, child sex, age, malaria, hemoglobin.

Just as in the continuous exposure models, child AFB_1_-lysine adduct concentration at 6 mo expressed as a dichotomous variable (detectable compared with nondetectable) was consistently negatively associated with HCZ at 6 mo (β = –0.41; 95% CI: –0.69, –0.12; *P* < 0·01), 12 mo (β = –0.28; 95% CI: –0.55, –0.01; *P* < 0·05), 18 mo (β = –0.33; 95% CI: –0.59, –0.08; *P* < 0·05), 24 mo (β = –0.29; 95% CI: –0.56, –0.03; *P* < 0·05) and 30 mo (β = –0.33; 95% CI: –0.63, –0.05; *P* < 0·05) (Table 5). However, the apparent difference in HCZ was evident among 1-wk-old infants, an outcome measured before the 6-mo exposure measure (Supplementary Figure 2). All significant associations between child AF exposure at 6 mo and HCZ at 6–30 mo were lost when we adjusted for 1 wk HCZ (Supplementary Figure 2). Maternal AF exposure in the third trimester was not correlated with child AF exposure at 6 mo (r_s_ = 0.14; *P* = 0.05).

**TABLE 5:** Association between aflatoxin B_1_-lysine adduct at 6 mo (detectable compared with nondetectable) and length-for-age *z*-score, weight-for-age *z*-score, weight-for-length *z*-score, MUAC for age *z*-score, and head circumference-for-age *z*-score, *n* = 170

Outcome assessment time point	Anthropometric outcome	Unadjusted^1^	Adjusted^1^^,^^2^
Anthropometry at 6 mo	Length-for-age *z*-score	–0.37 (–0.68, –0.05)	–0.32 (–0.66, 0.01)
	Weight-for-age *z*-score	–0.44 (–0.76, –0.11)∗	–0.44 (–0.77, –0.10)∗
	Weight-for-length *z*-score	–0.26 (–0.56, 0.05)	–0.22 (–0.53, 0.08)
	MUAC for age *z*-score	–0.37 (–0.67, –0.06)	–0.26 (–0.63, 0.10)
	Head circumference-for-age *z*-score	–0.48 (–0.76, –0.19)∗∗	–0.41 (–0.69, –0.12)∗∗
Anthropometry at 12 mo	Length-for-age *z*-score	–0.33 (–0.65, –0.01)∗	–0.28 (–0.6, 0.05)
	Weight-for-age *z*-score	–0.32 (–0.63, –0.01)∗	–0.30 (–0.61, 0.01)
	Weight-for-length *z*-score	–0.21 (–0.50, 0.07)	–0.23 (–0.51, 0.05)
	MUAC for age *z*-score	–0.10 (–0.39, 0.18)	–0.06 (–0.35, 0.23)
	Head circumference-for-age *z*-score	–0.28 (–0.55, –0.01)∗	–0.28 (–0.55, –0.01)∗
Anthropometry at 18 mo	Length-for-age *z*-score	–0.24 (–0.54, 0.05)	–0.20 (–0.49, 0.10)
	Weight-for-age *z*-score	–0.27 (–0.54, 0.00)	–0.28 (–0.55, –0.01)∗
	Weight-for-length *z*-score	–0.20 (–0.45, 0.05)	–0.18 (–0.43, 0.06)
	MUAC for age *z*-score	–0.12 (–0.38, 0.14)	–0.08 (–0.33, 0.17)
	Head circumference-for-age *z*-score	–0.32 (–0.58, –0.06)∗	–0.33 (–0.59, –0.08)∗
Anthropometry at 24 mo	Length-for-age *z*-score	–0.14 (–0.44, 0.17)	–0.15 (–0.45, 0.16)
	Weight-for-age *z*-score	–0.29 (–0.57, –0.02)∗	–0.34 (–0.61, –0.08)∗
	Weight-for-length *z*-score	–0.31 (–0.58, –0.03)∗	–0.36 (–0.61, –0.10)∗∗
	MUAC for age *z*-score	–0.26 (–0.52, 0.01)	–0.34 (–0.58, –0.10)∗∗
	Head circumference-for-age *z*-score	–0.28 (–0.55, –0.01)∗	–0.29 (–0.56, –0.03)∗
Anthropometry at 30 mo	Length-for-age *z*-score	–0.19 (–0.48, 0.11)	–0.19 (–0.49, 0.12)
	Weight-for-age *z*-score	–0.19 (–0.47, 0.08)	–0.15 (–0.43, 0.12)
	Weight-for-length *z*-score	–0.07 (–0.34, 0.20)	–0.07 (–0.34, 0.20)
	MUAC for age *z*-score	–0.16 (–0.42, 0.09)	–0.12 (–0.38, 0.14)
	Head circumference-for-age *z*-score	–0.36 (–0.64, –0.09)∗	–0.33 (–0.63, –0.05)∗

∗∗∗*P* < 0·001, ∗∗*P* < 0·01, ∗*P* <0·05.

1 β coefficient is the SD difference in the outcome per SD difference in log aflatoxin

2 Adjusted for maternal age, prepregnancy BMI, parity, zinc protoporphyrin, hemoglobin, malaria, HIV, weight and MUAC gain rate from baseline to 36 wk, food security, housing quality, season, child sex, age, porridge consumption, and predominant breastfeeding at 4 and 6 mo.

## Discussion

We evaluated the relationship between maternal AF B_1_-lysine adduct concentration (in utero and at 6 mo postpartum), child AF B_1_-lysine adduct concentration (at 6 and 18 mo of age), and child growth outcomes in the first 30 mo of life in a study population with a high risk of AF exposure. We found that in utero exposure was positively associated with birth size, whereas exposure in children at 6 and 18 mo was negatively associated with child growth outcomes in adjusted models.

AF is known as an important contributor to impaired child growth [25]. However, we observed a positive association between maternal AF exposure and birth size. This positive association was not expected, as most other studies have documented a negative association between AF exposure during pregnancy and newborn size [5,6,8]. For example, a study of 785 pregnant Ghanaian women showed that women with high serum AF (>11.34 pg⁄mg albumin) at delivery had significantly greater odds of having a low birth weight infant than those with low AF [6]. In Uganda, AF exposure measured in mid-pregnancy (18 wk) was associated with adverse birth outcomes, especially lower birth weight and smaller head circumference [8]. A Nepalese study reported a small but significant association between prenatal serum AFB_1_-lysine adduct concentrations and risk of a small for gestational age newborn, but not other birth outcomes [19]. The median serum AFB_1_-lysine adduct concentration reported in 2 of those 3 studies was lower than that seen in our study. The median maternal AFB_1_-lysine adduct concentration was 10.9 and 5.8 pg/mg albumin in the Ghana and Uganda studies and 1.4 pg/mg in the Nepalese study, respectively [6,8,19]. Women in our study had median values of 11.2 pg/mg albumin at baseline, 10.5 pg/mg albumin at 36 weeks of gestation, and 13.1 pg/mg albumin at 6 mo postpartum using a conversion to units of pg/mg albumin by multiplying values expressed in pg/μL by a factor of 23.8 [21].

There are several potential explanations for the positive association between prenatal exposure and birth size that we observed. One possibility is that maternal AF concentrations are positively associated with maternal total EI. Because maize comprises the staple food in this population and is a key source of AF exposure, the positive association between maternal AF concentration and birth outcomes could be because of a greater EI, primarily from maize, in this population. We attempted to adjust for EI during pregnancy using maternal weight at baseline, weight gain, and change in MUAC from baseline to 36 wk pregnancy as proxy variables. Maternal weight gain and change in MUAC during pregnancy are associated with EI [[26], [27], [28], [29], [30]]. Furthermore, to assess if we were including a variable on the causal pathway between maternal AF exposure and birth size, we considered HIV status and MUAC gain rate. However, the positive association between maternal AF concentration and birth size in our study remained significant after adjusting for these variables. Another possible explanation could be residual confounding by season. We adjusted for dry compared with the wet season, but still, there could be differences by month of birth (those having been in early pregnancy in the wet season would have been in late pregnancy during and right after harvest).

One other study conducted in Mexico noted a positive association between serum AFB_1_-lysine adduct concentrations in children and linear growth [20]. The exposure was assessed in children at around 8 mo of age, and the median AFB_1_-lysine adduct concentration was 0.82 pg/mg albumin, reflecting a low dose exposure. The authors hypothesized that their findings could be explained based on the theory of hormesis, which is defined as a dose-response phenomenon characterized by low-dose stimulation and high-dose inhibition. Hormesis is an adaptive strategy to optimize allocation of resources. It may occur using 2 different mechanisms of action that include direct stimulation and indirect stimulation. Both mechanisms are a result of overcompensation to maintain homeostasis following an initial disturbance. AFs, being acutely toxic to the liver, may lead to an overcompensation response in the animal that may ultimately present as a body mass increase [[31], [32], [33], [34], [35], [36], [37], [38]]. AF exposure has been shown to have a growth-promoting effect at low doses in chickens but a growth-inhibiting effect at high doses [31]. In contrast, in our study, it was maternal exposure during pregnancy and not child exposure at 6 and 18 mo that was positively associated with child growth. To our knowledge, our study is the first to document that high-dose maternal AF exposure during pregnancy was associated with a larger size at birth, but further research is needed to explain this finding.

Maternal postnatal exposure was equally high, as demonstrated by high AFB_1_-lysine adduct concentration, and was correlated with child exposure at 6 and 18 mo [21]. However, we did not find any significant association between maternal AF exposure at 6 mo postpartum and child growth outcomes. In this population, the maternal dietary staple, nsima (a maize-based meal), is similar to children’s main complementary food (a maize porridge), and so mother and child share a similar postnatal dietary source of AF. Maize accounts for >70% of daily EI in Malawi and children are mostly weaned on a maize-based porridge [39]. Contamination of maize by AF is high, with reports indicating AF concentrations almost twice the regulatory limit of 3 *μ*g/kg in Malawi [40]. AFB_1_-lysine reflects chronic exposure to AF, and it correlates well with AF intake from maize-based diets [41]. We were unable to find any documentation in previous literature on associations between maternal postnatal AF exposure and child growth.

Child AF exposure was negatively associated with child growth outcomes. HCZ was associated with AF exposure, and the magnitude of the association was greater than for other growth outcomes. The difference in mean HCZ between children with detectable and nondetectable AF at 6 mo was between 0.28 and 0.40 (Supplementary Figure 2). However, this HCZ deficit was already present at 1 wk of age and was substantial. The associations between HCZ and AF exposure became nonsignificant when we adjusted for HCZ at 1 wk, implying that it was exposed in utero that made the most difference, even though maternal AF was not related to smaller HCZ. We do not have adequate explanation for this finding based on reverse causality or controlling for factors that could be on the causal pathway, so further research is warranted to explore more. Interestingly maternal AF exposure measured in the third trimester was weakly associated with maternal AF exposure measured at 6 mo postpartum and was not associated with child AF exposure measured at 6 mo. Similarly, newborn size was not a predictor of infant feeding patterns. AF exposure in the first 6 mo occurs within a key period of rapid brain growth [42], so the persistent deficit in HCZ could have important consequences for development. We were unable to find other studies that have examined the effect of AF exposure on head circumference assessed at multiple time points. Using data from Uganda, Lauer et al. [8] found that high AF exposure in pregnancy was associated with smaller head circumference and lower HCZ at birth.

Child AFB_1_-lysine adduct concentration at 18 mo was negatively associated with nearly all of the child growth outcomes at 18 and 24 mo of age and with linear growth status at 30 mo of age. The negative association of child AF exposure with growth outcomes is consistent with what most studies have demonstrated [[9], [10], [11],^43^,^44^], and was observed within the time of peak incidence of growth faltering, the complementary feeding period [45]. We adjusted for a number of potential confounding factors similar to those included in previous studies, for example, child sex, season, indicators of socioeconomic status, breastfeeding status, and consumption of complementary food (porridge) at 4 and 6 mo. Additionally, we adjusted for maternal EI during pregnancy using a maternal weight at baseline, weight gain, and change in MUAC from baseline to 36 wk pregnancy as proxy variables.

How AF exposure may act to affect child growth is not well characterized. Potential mechanisms through which AF exposure may act to affect child growth include disruption of the IGF axis, environmental enteric dysfunction, and immunomodulation [[46], [47], [48], [49]]. Our results contrast somewhat with those observed in a randomly assigned trial studying the effects of reducing AF exposure in Kenya, where exposure is comparably high. The trial enrolled women at ≥20 weeks of gestation and followed their offspring at birth, 13 mo, and 22 mo of age. The intervention significantly reduced endline but not midline serum AFB_1_-lysine adduct concentrations. However, there was no improvement in child linear growth or stunting at the endline in the intervention group compared to the control group [50], even though the intervention increased LAZ at the midline by 0.16. Taken as a whole, the results of our study and others raise questions about the age at which AF exposure is most likely to adversely affect growth and the magnitude of exposure reduction required to see an impact on growth outcomes, especially in regions where exposure is very high.

The strengths of this study include a longitudinal design, from mid-pregnancy through 30 mo of age among the children, assessment of AF exposure at multiple time points in both the mothers and children and assessment of AF exposure using the gold standard method, IDMS. AFB_1_-lysine adduct has long-term stability in human cryopreserved blood samples stored at –80°C for over 15 y, and this has been assessed using IDMS [51]. We presented AFB_1_-lysine adduct concentrations in volumetric terms, pg/μL, without adjustment for albumin concentration because HSA normalization of AFB_1_-lysine values produces laboratory-dependent biases in estimates of AFB_1_ internal dose, and the quantitative bias between HSA assays can potentially lead to meaningful differences in statistical inference [52,53].

Exposure and outcome assessments were objective measures and, therefore, less susceptible to reporting bias. A limitation of this study is that we cannot ascertain causality because it is based on observational study data analysis. We adjusted for multiple potential confounders, yet there is still the possibility of residual confounding. Because we evaluated multiple outcomes, some of the associations could be due to chance. We did not conduct a post hoc adjustment for multiplicity because such correction was considered inappropriate [54]. There is also potential for selection bias since we included in our analysis only mother-child dyads for whom we had plasma samples.

In conclusion, child AF exposure is consistently negatively associated with child growth outcomes with a persistent deficit in HCZ during the first 2 and a half years of life. However, we find a significant positive association between maternal exposure during pregnancy and child growth outcomes, which is surprising. This highlights the complexity of the association between AF exposure and child growth. More studies are needed to examine this association and also to elucidate the mechanisms through which AF affects growth and possibly brain development.

## Author contributions

The authors’ responsibilities were as follows – AM, CDA, CPS, KGD: designed the research; PA, UA, KM, KGD: designed the main trial iLiNs-DYAD; PA, UA, KM, EK, DC, AM: conducted the iLiNs-DYAD trial; JWS, JDG: performed the laboratory analysis; AM: conducted the statistical analysis; CDA: advised on statistical analysis; AM, CPS: drafted the manuscript; PA, UA, KM, KGD, JWS, JDG, KJS, EK, DC: reviewed the draft manuscript, and all authors: read and approved the final manuscript.

## Funding

This study was supported by a grant from the Bill and Melinda Gates Foundation (BMGF) OPP1164205 issued to Johns Hopkins University. The iLiNS-DYAD (international lipid-based nutrient supplements) trial was funded by BMGF OPP49817 and FANTA (Food and Nutrition Technical Assistance III) project.

## Author disclosures

The authors report no conflicts of interest.

## Data availability

Data described in the manuscript, code book, and analytic code will be made available via a repository at the University of California-Davis for the iLiNS-DYAD studies and will be shared upon request pending the Principal Investigator’s approval.

## Declaration of Competing Interest

☒ The authors declare that they have no known competing financial interests or personal relationships that could have appeared to influence the work reported in this paper.
