# Supplementary material for: Child Aflatoxin Exposure is Associated with Poor Child Growth Outcomes: A Prospective Cohort Study in Rural Malawi
Source: Curr Dev Nutr. 2023 Jun 10;7(7):101962. doi: 10.1016/j.cdnut.2023.101962 (PMC10328803; doi:10.1016/j.cdnut.2023.101962)
Supplement: Multimedia component 1 [file mmc1.docx]

Online Supplementary Material

**Table of contents**

Supplementary Table 1: Aflatoxin B_1_-lysine adduct concentration

Supplementary Table 2: Association between maternal aflatoxin B_1_-lysine adduct concentration and child anthropometric status at 18, 24 and 30 months

Supplementary Table 3: List of potential covariates by anthropometric outcome assessment time point

Supplementary Figure 1: Comparison of Human Serum Albumin (HSA) assays

Supplementary Figure 2: Mean head circumference-for-age z-score (HCZ) from 1 week to 30 months by child aflatoxin exposure at 6 months

| **Supplementary Table 1. Aflatoxin B_1_-lysine adduct concentration** | | | | | |
| --- | --- | --- | --- | --- | --- |
|  | Baseline | 36 weeks gestation | 6 months postpartum | 6 months | 18 months |
| *Proportion of participants with detectable aflatoxin B_1_-lysine adduct concentration, % (n)* | | | | | |
| Mothers | 98% (230/235) | 96% (224/234) | 100% (220/220) |  |  |
| Children |  |  |  | 60% (122/204) | 95% (211/221) |
| *Aflatoxin B_1_-lysine adduct concentration (pg/µL) median (Q1, Q3)* | | | | | |
| Mothers | 0.47 (0.23, 0.97) | 0.44 (0.21, 1.07) | 0.55 (0.24, 1.14) |  |  |
| Children |  |  |  | 0.07 (0.01, 0.24) | 0.35 (0.19, 0.91) |

| **Supplementary Table 2.** **List of potential covariates by anthropometric outcome assessment time point** | | | | | | |
| --- | --- | --- | --- | --- | --- | --- |
| Variable | Variable assessment time point for each anthropometric outcome | | | | | |
|  | Newborn anthro. | Anthro. at 6 mo | Anthro. at 12 mo | Anthro. at 18 mo | Anthro. at 24 mo | Anthro. at 30 mo |
| Maternal age | Baseline | Baseline | Baseline | Baseline | Baseline | Baseline |
| Prepregnancy BMI | Baseline | Baseline | Baseline | Baseline | Baseline | Baseline |
| Parity | Baseline | Baseline | Baseline | Baseline | Baseline | Baseline |
| Maternal hemoglobin | Baseline |  |  |  |  |  |
| Maternal zinc protoporphyrin | Baseline |  |  |  |  |  |
| Maternal HIV status | Baseline | Baseline | Baseline | Baseline | Baseline | Baseline |
| Maternal malaria status | Baseline |  |  |  |  |  |
| Maternal weight gain rate from baseline to 36 weeks gestation | 36 weeks gestation | 36 weeks gestation | 36 weeks gestation | 36 weeks gestation | 36 weeks gestation | 36 weeks gestation |
| Maternal MUAC gain rate from baseline to 36 weeks gestation | 36 weeks gestation | 36 weeks gestation | 36 weeks gestation | 36 weeks gestation | 36 weeks gestation | 36 weeks gestation |
| Child sex | Birth | Birth | Birth | Birth | Birth | Birth |
| Child hemoglobin status |  | 6 mo | 12 mo | 18 mo | 24 mo | 30 mo |
| Child malaria status |  | 6 mo | 12 mo | 18 mo | 24 mo | 30 mo |
| Child zinc protoporphyrin |  | 6 mo | 12 mo | 18 mo | 24 mo | 30 mo |
| Porridge consumption |  | 4 and 6 mo |  |  |  |  |
| Predominant breastfeeding |  | 4 and 6 mo |  |  |  |  |
| Livestock asset | Baseline | Baseline | Baseline | Baseline | Baseline | Baseline |
| Household Food Insecurity Access | Baseline | 6 mo | 12 mo | 18 mo | 24 mo | 30 mo |
| Housing quality index | Baseline | Baseline | Baseline | Baseline | Baseline | Baseline |
| Season | Baseline | 6 mo | 12 mo | 18 mo | 24 mo | 30 mo |
| Intervention group | Baseline |  | 6 mo | 6 mo | 6 mo | 6 mo |

| **Supplementary Table 3.** **Association between maternal aflatoxin B_1_-lysine adduct concentration and length-for-age z-score, weight-for-age z-score, weight-for-length z-score, mid-upper-arm-circumference-for-age z-score, head circumference-for-age z-score at 18, 24 and 30 months, n =[β^1^ (95% CI)]** | | | | | | |
| --- | --- | --- | --- | --- | --- | --- |
|  | Maternal aflatoxin B_1_-lysine adduct | | | | | |
|  | Baseline | | 36 weeks | | 6 months postpartum | |
|  | Unadjusted | Adjusted^2^ | Unadjusted | Adjusted^2^ | Unadjusted | Adjusted^2^ |
|  | β (95% CI) | β (95% CI) | β (95% CI) | β (95% CI) | β (95% CI) | β (95% CI) |
| *Anthropometric outcomes at 18 months, n=207* | | | | | | |
| LAZ | 0.07 (-0.06, 0.21) | 0.05 (-0.09, 0.19) | 0.13 (0.00, 0.26)* | 0.12 (-0.01, 0.26) | -0.02 (-0.16, 0.12) | -0.03 (-0.17, 0.11) |
| WAZ | 0.01 (-0.12, 0.13) | 0.02 (-0.10, 0.15) | 0.06 (-0.06, 0.19) | 0.07 (-0.05, 0.20) | -0.02 (-0.15, 0.11) | -0.04 (-0.16, 0.09) |
| WLZ | -0.04 (-0.16, 0.07) | -0.01 (-0.12, 0.11) | -0.01 (-0.12, 0.11) | 0.01 (-0.11, 0.12) | -0.02 (-0.14, 0.10) | -0.01 (-0.13, 0.11) |
| MUACZ | 0.04 (-0.08, 0.16) | 0.07 (-0.05, 0.19) | 0.05 (-0.06, 0.17) | 0.05 (-0.07, 0.16) | 0.06 (-0.06, 0.18) | 0.06 (-0.06, 0.17) |
| HCZ | -0.03 (-0.15, 0.09) | -0.03 (-0.15, 0.09) | 0.01 (-0.11, 0.13) | 0.02 (-0.10, 0.14) | -0.05 (-0.17, 0.07) | -0.07 (-0.20, 0.05) |
| *Anthropometric outcomes at 24 months, n=187* | | | | | | |
| LAZ | 0.03 (-0.12, 0.17) | 0.00 (-0.14, 0.14) | 0.06 (-0.09, 0.20) | 0.07 (-0.08, 0.22) | -0.03 (-0.17, 0.12) | -0.02 (-0.17, 0.12) |
| WAZ | -0.04 (-0.17, 0.10) | -0.01 (-0.14, 0.12) | 0.05 (-0.08, 0.19) | 0.10 (-0.04, 0.23) | -0.04 (-0.18, 0.09) | -0.06 (-0.18, 0.07) |
| WLZ | -0.07 (-0.20, 0.06) | -0.03 (-0.16, 0.09) | 0.03 (-0.10, 0.16) | 0.07 (-0.06, 0.20) | -0.04 (-0.17, 0.09) | -0.06 (-0.18, 0.07) |
| MUACZ | -0.01 (-0.14, 0.12) | 0.00 (-0.12, 0.12) | 0.03 (-0.10, 0.16) | 0.06 (-0.06, 0.19) | 0.01 (-0.12, 0.14) | -0.02 (-0.14, 0.10) |
| HCZ | -0.06 (-0.19, 0.07) | -0.07 (-0.20, 0.06) | -0.06 (-0.19, 0.07) | -0.05 (-0.18, 0.08) | -0.08 (-0.21, 0.05) | -0.10 (-0.23, 0.03) |
| *Anthropometric outcomes at 30 months, n=179* | | | | | | |
| LAZ | -0.02 (-0.16, 0.12) | -0.04 (-0.18, 0.10) | 0.12 (-0.03, 0.26) | 0.10 (-0.05, 0.25) | -0.05 (-0.19, 0.10) | -0.05 (-0.19, 0.10) |
| WAZ | -0.08 (-0.21, 0.05) | -0.06 (-0.19, 0.06) | 0.04 (-0.10, 0.17) | 0.02 (-0.11, 0.16) | -0.03 (-0.17, 0.10) | -0.03 (-0.17, 0.10) |
| WLZ | -0.12 (-0.25, 0.01) | -0.06 (-0.19, 0.07) | -0.05 (-0.18, 0.09) | -0.04 (-0.17, 0.09) | -0.01 (-0.14, 0.12) | 0.00 (-0.13, 0.13) |
| MUACZ | 0.00 (-0.12, 0.13) | 0.01 (-0.11, 0.13) | 0.02 (-0.10, 0.15) | 0.03 (-0.10, 0.16) | 0.00 (-0.13, 0.12) | 0.01 (-0.12, 0.13) |
| HCZ | -0.11 (-0.25, 0.02) | -0.13 (-0.26, 0.01) | -0.08 (-0.21, 0.06) | -0.07 (-0.20, 0.07) | -0.11 (-0.24, 0.03) | -0.10 (-0.23, 0.04) |

***P <0·001, **P < 0·01, *P <0·05

1. β coefficient is standard deviation difference in outcome per standard deviation difference in log aflatoxin

2. Adjusted for maternal pre-pregnancy BMI, HIV, weight and MUAC gain rate from baseline to 36 weeks, housing quality, food security, child sex, age, malaria, hemoglobin

LAZ = Length-for-age z-score, WAZ = Weight-for-age z-score, WLZ = Weight-for-length z-score, MUACZ = Mid-upper-arm-circumference-for-age z-score, HCZ = Head circumference-for-age z-score

**Supplementary Figure 1: Comparison of Human Serum Albumin (HSA) assays**


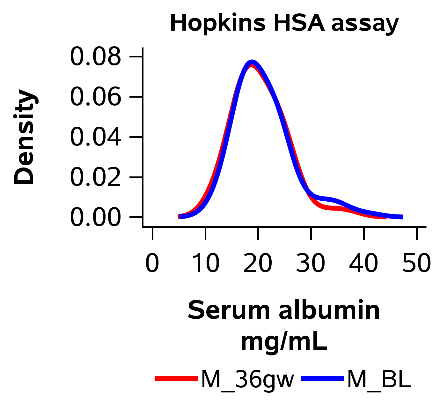

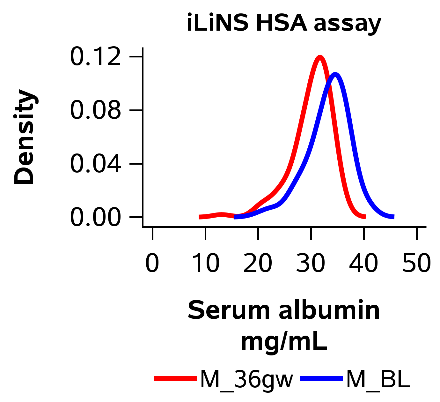

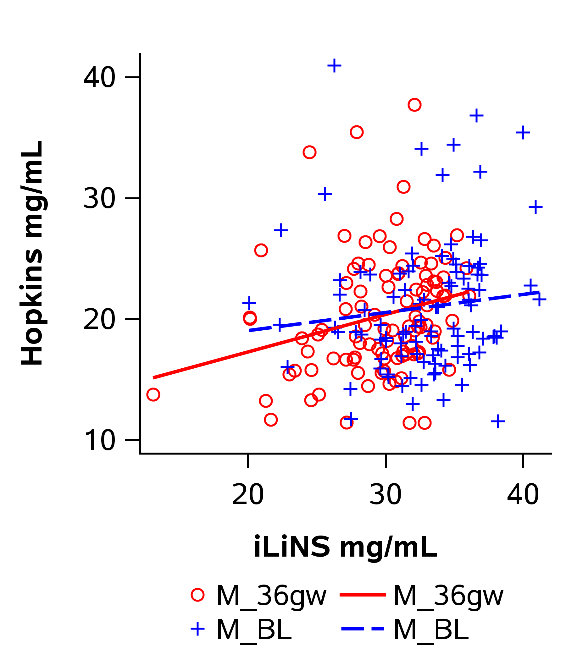


**Supplementary Figure 2: Mean head circumference-for-age z-score (HCZ) from 1 week to 30 months by aflatoxin exposure in child at 6 months (detectable vs non-detectable aflatoxin)**

Note: Figure above describes mean head circumference-for-age z-score (HCZ) from 1 week to 30 months in children with detectable (red line) and non-detectable (blue line) aflatoxin exposure at 6 months This is adjusted for maternal HIV status, zinc protoporphyrin, mid-upper-arm-circumference (MUAC) gain rate from baseline to 36 weeks, season, child malaria and hemoglobin, and intervention group.

All significant associations between HCZ 6-30 months and aflatoxin exposure (detectable vs. non-detectable) at 6 months were lost when we adjusted for HCZ at 1 week in the models (β = -0.20; 95% CI: -0.45, 0.04, β = -0.02; 95% CI: -0.25, 0.21, β = -0.08; 95% CI: -0.31, 0.14, β = -0.04; 95% CI: -0.29, 0.20, β = -0.12; 95% CI: -0.39, 0.15 at 6, 12, 18, 24 and 30 months respectively)
